# From the Liver to Heart in Nonalcoholic Fatty Liver Disease: Single‐Cell and Serum Evidence for Cytokeratin 18 in Predicting Cardiovascular Risk

**DOI:** 10.1155/cdr/3055531

**Published:** 2026-01-15

**Authors:** Hongjing Xu, Xiang Ma, Fengjuan Chen, Bin Zhou

**Affiliations:** ^1^ Department of Gastroenterology, Wuxi Xishan People′s Hospital, Wuxi, Jiangsu Province, China

**Keywords:** cardiovascular disease, cytokeratin, fatty liver disease, prediction, serum

## Abstract

**Background:**

This study is aimed at measuring the expression of serum cytokeratin 18 (CK18) in patients with nonalcoholic fatty liver disease (NAFLD) and at evaluating its ability to discriminate NAFLD patients with and without coronary heart disease (CHD), thereby elucidating its potential role in reflecting the link between NAFLD and increased cardiovascular involvement.

**Methods:**

A total of 127 patients diagnosed with NAFLD and treated between September 2022 and July 2024 were enrolled in this cross‐sectional study. Based on the presence or absence of CHD, they were divided into two subgroups: a CHD‐present group (*n* = 79) and a CHD‐absent group (*n* = 48). In addition, a control group consisting of 100 age‐matched and sex‐matched healthy individuals undergoing routine physical examinations during the same period was included for comparison.

**Results:**

Compared with the control group, patients with NAFLD showed significantly higher levels of alanine aminotransferase (ALT), serum CK18‐M65 and CK18‐M30 levels, body mass index (BMI), homeostasis model assessment of insulin resistance index (HOMA‐IR), triglyceride (TG), total cholesterol (TC), aspartate aminotransferase (AST), and waist‐to‐hip ratio (*p* < 0.05). Multivariate logistic regression analysis identified serum CK18‐M65, CK18‐M30, TC, ALT, AST, and HOMA‐IR as independent factors in NAFLD patients (*p* < 0.05). The combination of CK18‐M65 and CK18‐M30 yielded an area under the curve of 0.843, with a sensitivity of 87.34% and a specificity of 75.00%. Functional enrichment analysis revealed that CK18‐related genes were involved in inflammatory signaling and cardiovascular regulatory pathways, supporting a mechanistic role for CK18 in the liver–heart axis.

**Conclusion:**

Serum CK18 may serve as a useful biomarker for identifying NAFLD patients with concurrent CHD, offering diagnostic value in clinical assessment.

## 1. Introduction

Nonalcoholic fatty liver disease (NAFLD), recently redefined as metabolic dysfunction–associated steatotic liver disease (MASLD), represents a spectrum of liver injury attributable to metabolic stress and is strongly associated with genetic susceptibility and insulin resistance, although its pathological changes are unrelated to alcohol [1, 2]. Although the updated MASLD/MASH nomenclature better reflects the metabolic underpinnings of the disease, the term NAFLD is retained in this study for consistency with previous CK18‐related literature.

In China, the prevalence of NAFLD has been rising steadily, affecting approximately 20%–30% of the general population and reaching even higher rates among individuals with metabolic disorders such as obesity and diabetes. Beyond hepatic injury, NAFLD is now recognized as a multisystem disorder that is closely linked to cardiovascular diseases (CVDs), diabetes mellitus, and other chronic diseases [3]. The disease can progress from simple steatosis to fibrosis, cirrhosis, or even liver failure, posing a significant health burden [4, 5]. Although NAFLD is a known risk factor for hepatocellular carcinoma and end‐stage liver disease, most NAFLD‐related deaths result from CVD, accounting for over 60% of total mortality [6].

The mechanistic link between NAFLD and CVD involves several interrelated pathways, including insulin resistance, inflammation responses, oxidative stress, epicardial fat accumulation and endothelial dysfunction. Among these, insulin resistance serves as a central feature, altering systemic metabolism and increasing cardiovascular risk [7]. Inflammatory cytokines released from the liver may further promote atherosclerosis [8], whereas oxidative stress exacerbates both hepatic injury and vascular damage [9]. In addition, epicardial fat accumulation secretes pro‐inflammatory and procoagulant factors that accelerate atherosclerosis and CHD progression [10].

Though liver biopsy remains the gold standard for diagnosing NAFLD, its clinical use is limited due to its invasive nature and associated complications. Cytokeratin 18 (CK18), a crucial intermediate filament protein expressed in epithelial cells of the liver, heart, and other organs is released into the circulation in two major forms during hepatocellular apoptosis and necrosis, appearing as two measurable serum fragments: the intact form (M65) and the caspase‐cleaved one (M30) [11]. Serum CK18 levels have been shown to correlate with hepatocyte apoptosis and fibrosis severity, distinguishing early from advanced NAFLD stages [12].

Previous studies have primarily focused on CK18 as a biomarker of steatohepatitis (NASH/MASH) and fibrosis progression [13, 14], but its potential association with cardiovascular involvement in NAFLD remains largely unexplored. Given that both hepatic and cardiac tissues express CK18, this study is aimed at investigating whether serum CK18 reflects concurrent cardiovascular alterations in NAFLD and at examining its mechanistic relevance through bioinformatics and single‐cell analyses.

Specifically, we evaluated the discriminatory performance of serum CK18‐M30 and CK18‐M65 for identifying NAFLD patients with CHD and explored the molecular plausibility of a CK18‐mediated liver–heart axis. These findings are expected to provide novel insights into the role of CK18 in linking hepatic injury to cardiovascular dysfunction, thereby informing strategies for early diagnosis and management of cardiovascular complications in NAFLD/MASLD.

## 2. Methods

### 2.1. Single‐Cell RNA Sequencing (scRNA‐seq) Data Analysis of CK18 Distribution

The scRNA‐seq datasets of human liver and heart tissues were obtained from the Tabula Sapiens database (https://tabula-sapiens-portal.ds.czbiohub.org). This dataset was selected for its comprehensive cross‐tissue annotation and consistent processing pipeline, minimizing batch variability across organs. Processed AnnData‐formatted files (h5ad) containing normalized expression matrices and cell annotations (cell types and subpopulations) were directly obtained from the database.

All preprocessing and normalization procedures were performed using Python 3.9.15, Scanpy (v1.9.1), and pandas (v1.5.3). To control for potential batch effects, datasets were integrated and normalized using the Scanpy function and (log1p(CPM+1)) transformation. The expression data for CK18 (KRT18_ENSG00000111057) was extracted and integrated with the corresponding cell‐type annotations for subsequent analysis.

Dimensionality reduction was performed using Uniform Manifold Approximation and Projection (UMAP in Scanpy, with default parameters). The spatial distribution of CK18 expression across different cell populations was visualized by UMAP heatmaps using Matplotlib (v3.6.2). To further compare CK18 expression across liver and heart cell populations, violin plots were generated using the Seaborn (v0.12.2) library in Python.

### 2.2. Cell–Cell Communication Analysis Between Liver and Cardiac Cells

For cell–cell communication analysis, the expression matrix (expr_matrix.csv) and corresponding cell metadata (cell_meta.csv) were extracted from the preprocessed data obtained from the Tabula Sapiens dataset. Expression profiles of KRT18^high hepatocytes and key cardiac populations (cardiomyocytes, fibroblasts, and endothelial cells) were used to create a CellChat object in R (v4.2.0) using the CellChat package (v1.6.1).

### 2.3. Prediction of Ligand–Receptor Interactions

CellChat analysis was performed with standard statistical thresholds to identify significant ligand–receptor interactions (adjusted *p* < 0.05, Benjamini–Hochberg FDR correction). Human ligand–receptor interaction databases integrated within CellChat were used as references. Cell–cell communication probabilities were computed without stringent significance filtering (raw.use = TRUE). This approach has been replaced with FDR‐controlled filtering to reduce false positives and retain only significant communication probabilities. To contextualize our findings, we additionally examined the Human Protein Atlas (HPA) Single Cell/Single Cell Type reference (https://www.proteinatlas.org/humanproteome/single+cell; accessed July 2025). Expression of key ligand–receptor genes (e.g., TGFB1, IL6, TGFBR2, and IL6R) was queried in the HPA single‐cell database.

### 2.4. Validation of Candidate Ligand–Receptor Expression

When significant communication probabilities were not detected, the expression levels of specific ligands (TGFB1, TGFB2, TGFB3, IL6, and CXCL10) in KRT18^high hepatocytes and their corresponding receptors (TGFBR2, IL6R, and CXCR3) in cardiac populations were examined to assess biological plausibility rather than infer direct signaling. The mean log‐normalized expression levels for each gene were calculated using rowMeans() in R. Violin plots and boxplots were generated using ggplot2 (v3.3.6) to visualize gene expression distributions. These analyses were considered exploratory and descriptive, aimed at identifying potential liver–heart communication axes for future validation.

### 2.5. Pathway Analysis

To the potential mechanisms underlying CK18 elevation in the context of NAFLD and CVD, pathway‐based bioinformatics analysis was performed using the KEGG database (https://www.genome.jp/kegg/pathway.html). Pathways such as “NAFLD,” “TNF signaling,” “NF‐*κ*B signaling,” and “insulin signaling” were analyzed to elucidate the potential involvement of CK18 in metabolic dysregulation, inflammation, and apoptosis. These analyses were intended to provide biological context rather than infer causality.

### 2.6. Subjects

In this cross‐sectional study, 127 patients diagnosed with NAFLD and treated at our hospital from September 2022 to July 2024 were enrolled as the fatty liver disease cohort. According to the presence or absence of CHD, they were further divided into a CHD‐absent group (*n* = 48) and a CHD‐present group (*n* = 79). In addition, 100 age‐matched and sex‐matched healthy individuals who underwent physical examination during the same period were randomly included as the control group. All CHD cases were preexisting diagnoses confirmed prior to enrollment, rather than newly developed during the study. The study was approved by the institutional ethics committee of our hospital, and all participants provided written informed consent.

### 2.7. Inclusion and Exclusion Criteria

The following inclusion criteria were employed: (1) patients who met the diagnostic criteria for NAFLD according to the “Guidelines for the Diagnosis and Treatment of Nonalcoholic Fatty Liver Disease” [15], confirmed by imaging (ultrasound, CT, or MRI); (2) patients with CHD who fulfilled the “Guidelines for the Diagnosis and Treatment of Chronic Coronary Syndrome” [16], including clinical manifestations of myocardial ischemia such as exertional chest pain or tightness; objective evidence of coronary atherosclerotic stenosis confirmed by coronary angiography (≥ 50% luminal narrowing in at least one major coronary artery), or by CT coronary angiography (CCTA); and supportive findings on electrocardiogram (ECG), exercise stress testing, or myocardial perfusion imaging consistent with ischemic heart disease; (3) patients with normal cognitive function who could understand and cooperate with study procedures; and (4) voluntary participation after being informed about the study protocol. The exclusion criteria involved (1) presence of liver cirrhosis hepatocellular carcinoma, or prior liver transplantation; (2) history of cardiac surgery or congenital heart disease; (3) viral hepatitis, alcoholic liver disease, autoimmune liver disease, or drug‐induced liver injury; (4) heavy alcohol intake (> 140 g ethanol/week) or use of hepatotoxic drugs within the past 3 months; (5) severe chronic systemic diseases such as advanced diabetes mellitus, uncontrolled hypertension, malignant tumors, or severe renal/cardiac insufficiency; and (6) use of lipid‐lowering or insulin‐sensitizing drugs within 3 months before enrollment. In addition, patients with diabetes mellitus and hypertension were excluded to avoid potential confounding effects, as both conditions may independently alter serum CK18 levels, systemic inflammation, and lipid metabolism, which could obscure the specific relationship between NAFLD and CHD.

### 2.8. Sample Collection

Blood (5 mL) was harvested from the veins of each fasted subject in the morning, followed by 15 min of centrifugation in a low‐temperature high‐speed centrifuge (TG16B, Hangzhou Chuanheng Experimental Instrument Co., Ltd.) at 4500 rpm. The acquired supernatant was stored in an ultralow temperature refrigerator (BDF‐86 V348, Jinan Jiangxue Medical Appliance Co., Ltd.) at −80°C for subsequent detection.

### 2.9. Evaluation of Outcomes

General demographic and clinical data of the participants, including body mass index (BMI), gender, waist‐to‐hip ratio, and age were recorded.

The levels of aspartate aminotransferase (AST), triglycerides (TGs), total cholesterol (TC), albumin (ALB), total bilirubin (TBIL), and alanine aminotransferase (ALT) were measured using an automatic biochemical analyzer (BS‐280, Vedeng Medical Co., Ltd.). For sample preparation, all subjects fasted for at least 12 h before blood collection and were asked to rest in a sitting position for ≥ 5 min to minimize posture‐related bias. Then, the same 5 mL fasting venous blood sample was collected into an EDTA anticoagulation tube through venipuncture and centrifuged for 15 min within 30 min of collection to separate plasma. All serum samples were aliquoted immediately, stored at −80°C, and thawed only once before assay to avoid degradation. Each sample was processed within 2 h after removal from the freezer to ensure sample stability. The obtained plasma was analyzed following standard enzyme‐linked immunoassay procedures. Briefly, 100 *μ*L of diluted reference serum and 100 *μ*L of each sample were added into the corresponding wells, with diluent alone added into blank wells. The plates were incubated for 1 h at 37°C, washed three times with buffer, and subsequently incubated with enzyme‐labeled antibodies (100 *μ*L/well, dilution dependent on antibody titer, e.g., 1:20,000) for another 1 h at 37°C. Afterwards, substrate buffer (100 *μ*L in volume) was added to each well and incubated at room temperature for 15 min in the dark. The reaction was stopped by adding 50 *μ*L of stop solution, and the optical density (OD) was measured at 450 nm using a microplate reader. The concentrations of the respective biochemical indicators were calculated based on standard curves. Additionally, the homeostasis model assessment of insulin resistance (HOMA‐IR) was calculated using the following formula: HOMA‐IR = (fasting blood glucose [FBG] × fasting insulin [FINS]) ÷ 22.5.

Enzyme‐linked immunosorbent assay was performed to determine the serum concentrations of CK18‐M65 and CK18‐M30. Briefly, standards were serially diluted using the provided standard diluent, 50 *μ*L of each diluted standard or serum sample was added into the respective wells of a precoated microplate. A blank well without sample or enzyme conjugate was included as a negative control. Following the manufacturer′s instructions, biotinylated anti‐CK18‐M30 antibody and HRP‐labeled avidin were sequentially added to the wells. Plates were incubated at room temperature for 2 h, then thoroughly washed to remove unbound components. The wash buffer was diluted 1:50 with distilled water prior to use. Afterwards, 3,3′,5,5′‐tetramethylbenzidine (TMB) substrate solution was added and incubated at 37°C for 15 min, during which the solution turned blue under peroxidase activity and yellow after acid termination. The reaction was stopped by adding 50 *μ*L of stop solution and the OD was measured at 450 nm using a microplate reader. The concentrations of CK18‐M65 and CK18‐M30 were determined from the standard curves. Both CK18‐M65 and CK18‐M30 ELISA kits demonstrated adequate analytical performance, with high linearity across the calibration range and intra‐assay /interassay coefficients of variation below 10%.

All samples were analyzed in duplicate, and the mean value was used for statistical analysis. Laboratory personnel were blinded to the CHD status of participants. According to the manufacturer′s specifications, the CK18‐M65 and CK18‐M30 ELISA kits had a limit of detection (LoD) of 0.1 U/mL, a linear range of 0.1–20 U/mL, and intra‐assay /interassay coefficients of variation < 8% and< 10%, respectively. The assays demonstrated good linearity (*R* ^2^ > 0.99) across the tested range.

The kits (Cat. No.: SBJ‐H0288 for CK18‐M65 and SBJ‐H0287 for CK18‐M30) were bought from Nanjing SenBeiJia Biological Technology Co., Ltd.

### 2.10. Statistical Analysis

Statistical analyses were performed using SPSS 23.0 software. Measurement data (CK18‐M65, CK18‐M30, etc.) were expressed as mean ± standard deviation ($\overset{¯}{x}\pm s$). The normality of data distribution was assessed using the Shapiro–Wilk test. Variables conforming to a normal distribution were compared between groups using the independent‐samples *t*‐test, whereas those not normally distributed were analyzed with the Mann–Whitney *U* test. Where appropriate, log transformation was considered to approximate normality. Count data were compared using *χ* ^2^ test and expressed as percentage (n [%]). For NAFLD subjects, potential contributing factors for CHD were identified through multivariate logistic regression analysis. The discriminatory ability of serum CK18 was assessed by plotting receiver operating characteristic (ROC) curves. The combined CK18 index (“Combination”) was derived from a binary logistic regression model using both CK18‐M30 and CK18‐M65 as predictors: logit(P) = *β*0 + *β*1(CK18 − M30) + *β*2 (CK18‐M65), where P represents the predicted probability of CHD. The area under the curve (AUC) and 95% confidence intervals were calculated using the DeLong method. Internal validation was performed with 1,000 bootstrap resamples to correct for optimism. Calibration performance was assessed by plotting predicted versus observed probabilities and by estimating calibration intercept and slope. Clinical utility was evaluated by decision curve analysis, comparing the net benefit of the combined model with those of single markers and “treat‐all”/“treat‐none” strategies. Given the exploratory nature of this study, no adjustment for multiple comparisons was applied. *p* < 0.05 indicated statistical significance.

## 3. Results

### 3.1. Single‐Cell Distribution of CK18 in Human Liver and Heart Tissues

scRNA‐seq analysis from the Tabula Sapiens database were revealed distinct CK18 expression patterns across cell types (Figure 1a,b and 2a,b). In the liver dataset, CK18 expression was predominantly enriched in hepatocytes and cholangiocytes (biliary epithelial cells), whereas in the heart dataset, CK18 was mainly detected in mesothelial and endothelial cells, with lower levels in cardiomyocyte. These results support a consistent tissue‐specific expression profile of CK18, suggesting its enrichment in hepatic parenchymal cells and moderate presence in certain cardiac populations, providing a plausible cellular framework for liver–heart crosstalk.

Figure 1CK18 expression in human liver scRNA‐seq dataset. (a) UMAP visualization of CK18 expression and (b) violin plots comparing CK18 expression levels across liver cell types.

**Figure:**
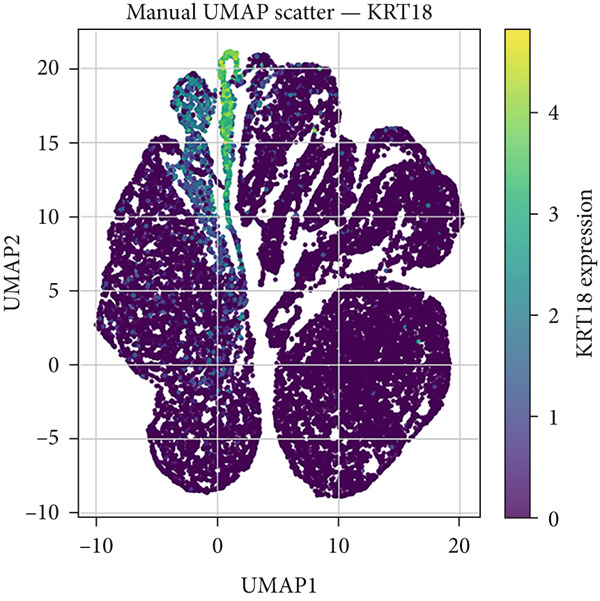
(a)

**Figure:**
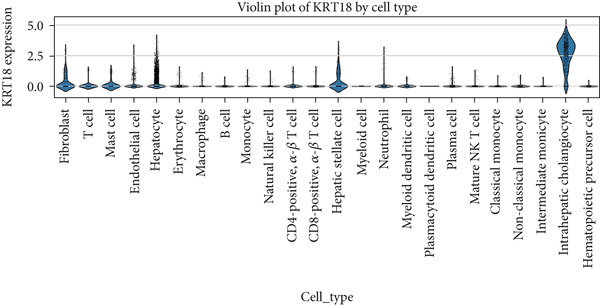
(b)

Figure 2CK18 expression in human heart scRNA‐seq dataset. (a) UMAP visualization of CK18 expression and (b) violin plots comparing CK18 expression levels across cardiac cell types.

**Figure:**
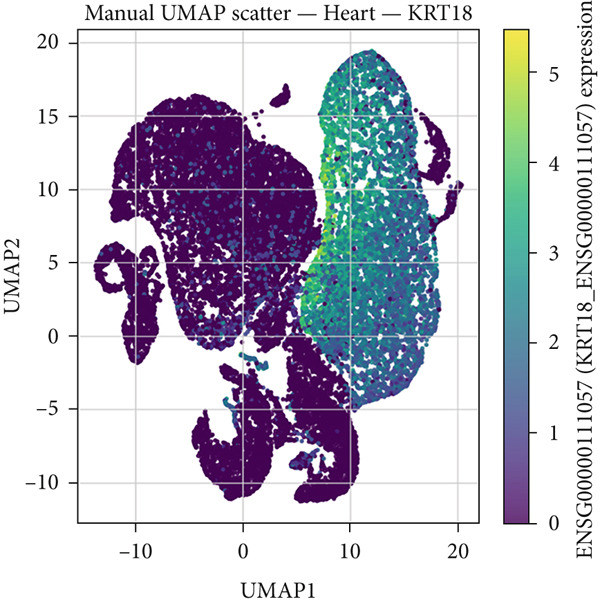
(a)

**Figure:**
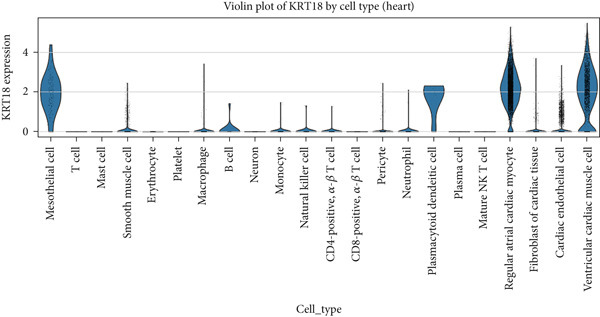
(b)

### 3.2. Analysis of Cell–Cell Communication in the Liver–Heart Axis

**Figure 3:**
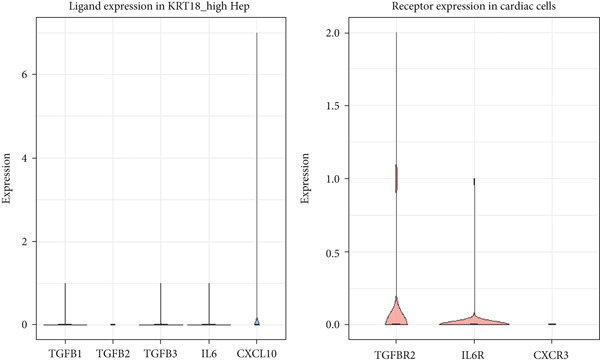
Expression of ligand (KRT18^high hepatocytes) and receptor (cardiac cells) gene pairs potentially involved in liver–heart axis communication.

Based on the identified cellular distribution of CK18, CellChat predicted several potential ligand–receptor pairs between KRT18^high hepatocytes and cardiac cells. After FDR correction (adjusted *p* < 0.05), only weak or moderate communication probabilities were retained, primarily involving TGF‐*β* and IL‐6 pathways. Expression levels of candidate ligands (TGFB1, TGFB2, TGFB3, IL6, and CXCL10) in hepatocytes and their corresponding receptors (TGFBR2, IL6R, and CXCR3) in cardiac cells were low but detectable (mean log‐normalized range 0.0015–0.034 for ligands and 0.01–0.08 for receptors) (Figure 3, Table S1). Reference analysis using the HPA single‐cell resource revealed similar expression patterns. TGFB1 was detected in multiple cell types, and IL6 exhibited generally low expression across most cell types, which aligned with our CellChat results. Collectively, these findings suggest that KRT18^high hepatocytes have the molecular potential to modulate cardiac cell populations via TGF‐*β* and IL‐6 signaling axes, thus providing biological plausibility for the proposed liver–heart axis hypothesis.

### 3.3. Pathway Analysis Results

Pathway enrichment analysis linked CK18 elevation to processes involved in NAFLD pathogenesis, including insulin resistance, TNF‐*α*‐mediated inflammation, and apoptosis (Figure S1), which supported the biological plausibility of CK18 participation in systemic metabolic and inflammatory pathways.

### 3.4. Serum CK18 Levels in Fatty Liver Disease and Control Groups

Serum levels of CK18‐M65 and CK18‐M30 were significantly higher in the fatty liver disease group than in the control group (*p* < 0.05) (Table 1).

**Table 1:** Levels of serum CK18 in fatty liver disease and control groups ($\overset{¯}{x}\pm s$).

**Group**	**n**	**CK18-M65 (U/mL)**	**CK18-M30 (U/mL)**
Control	100	72.63 ± 18.50	41.69 ± 10.82
Fatty liver disease	127	149.47 ± 36.42	103.79 ± 36.29
*t*		19.229	16.536
*p*		< 0.001	< 0.001

### 3.5. Clinical Data of Subjects in Control and Fatty Liver Disease Groups

No statistically significant differences were observed between the fatty liver disease and control groups in terms of sex ratio, age, and ALB and TBIL levels (*p* > 0.05). In contrast, the fatty liver disease group exhibited significantly higher BMI, waist‐to‐hip ratio, TC, TG, ALT, AST, and HOMA‐IR compared with the control group (*p* < 0.05) (Table 2).

**Table 2:** Clinical data of subjects in control and fatty liver disease groups (*n* [%], $\overset{¯}{x}\pm s$).

**Group**	**Control (** **n** = 100**)**	**Fatty liver disease (** **n** = 127**)**	**t**/**χ** ^2^	**p**
Gender (%)			2.202	0.138
Female	41 (41.00)	40 (31.50)		
Male	59 (59.00)	87 (68.50)		
Age (year)	42.69 ± 6.09	41.16 ± 12.75	1.105	0.271
BMI (kg/m^2^)	20.58 ± 1.60	27.85 ± 4.27	16.150	< 0.001
Waist‐to‐hip ratio	0.86 ± 0.05	0.94 ± 0.16	4.816	< 0.001
TC (mmol/L)	4.82 ± 0.61	5.15 ± 1.10	2.691	0.008
TG (mmol/L)	0.93 ± 0.30	2.85 ± 0.73	24.701	< 0.001
ALB (g/L)	41.59 ± 5.12	42.64 ± 4.86	1.578	0.116
TBIL (*μ*mol/L)	12.16 ± 6.82	13.50 ± 5.19	1.681	0.094
ALT (U/L)	18.49 ± 5.42	65.83 ± 21.69	21.299	< 0.001
AST (U/L)	29.75 ± 5.30	56.25 ± 17.68	14.479	< 0.001
HOMA‐IR	1.79 ± 0.47	3.64 ± 1.11	15.597	< 0.001

### 3.6. Results of Univariate Analysis on Serum CK18 Levels in CHD‐Present and CHD‐Absent Groups

Univariate analysis showed that serum CK18‐M65 and CK18‐M30 levels were significantly higher in the CHD‐present group than in the CHD‐absent group (*p* < 0.05) (Table 3).

**Table 3:** Results of univariate analysis on serum CK18 levels in CHD‐present and CHD‐absent groups ($\overset{¯}{x}\pm s$).

**Group**	**n**	**CK18-M65 (U/mL)**	**CK18-M30 (U/mL)**
CHD‐absent	48	153.63 ± 32.77	92.61 ± 27.49
CHD‐present	79	173.85 ± 46.80	112.85 ± 36.90
*t*		2.626	3.285
P		0.010	0.001

### 3.7. Results of Univariate Analysis on Clinical Data From Subjects in CHD‐Present and CHD‐Absent Groups

Univariate analysis revealed no statistically significant differences in sex ratio, waist‐to‐hip ratio, age, BMI, TG, ALB, and TBIL levels between the two groups (*p* > 0.05). In contrast, the CHD‐present group exhibited significantly higher TC, ALT, AST, and HOMA‐IR levels compared with the CHD‐absent group (*p* < 0.05) (Table 4).

**Table 4:** Results of univariate analysis on clinical data from subjects in CHD‐present and CHD‐absent groups (*n* [%], $\overset{¯}{x}\pm s$).

**Group**	**CHD-absent (** **n** = 48**)**	**CHD-present (** **n** = 79**)**	**t**/**χ** ^2^	**p**
Gender (%)			1.289	0.256
Female	18 (37.50)	22 (27.85)		
Male	30 (62.50)	57 (72.15)		
Age (year)	44.65 ± 12.00	45.49 ± 8.76	0.454	0.650
BMI (kg/m^2^)	28.32 ± 3.58	27.79 ± 2.50	0.981	0.329
Waist‐to‐hip ratio	0.91 ± 0.18	0.93 ± 0.21	0.549	0.584
TC (mmol/L)	4.74 ± 1.19	5.58 ± 1.41	3.447	0.001
TG (mmol/L)	1.79 ± 0.85	2.04 ± 1.38	1.131	0.260
ALB (g/L)	41.96 ± 10.25	42.82 ± 7.84	0.533	0.595
TBIL (*μ*mol/L)	14.35 ± 4.73	13.07 ± 5.47	1.344	0.181
ALT (U/L)	39.74 ± 11.36	76.52 ± 22.83	10.396	< 0.001
AST (U/L)	31.90 ± 13.91	65.14 ± 16.81	11.509	< 0.001
HOMA‐IR	2.35 ± 0.68	2.80 ± 0.75	3.394	0.001

### 3.8. Multivariate Logistic Regression Analysis Results of NAFLD Subjects for Factors Contributing to CHD

A multivariate logistic regression analysis was performed with CHD (presence or absence) as the dependent variable and the variables showing statistical significance in the univariate analyses as independent predictors. To improve interpretability, CK18‐M30 and CK18‐M65 values were rescaled per 10 U/mL increase before analysis. The results indicated that each 10 U/mL increase in CK18‐M30 and CK18‐M65 was independently associated with higher odds of CHD, along with elevated TC, ALT, AST, and HOMA‐IR levels (*p* < 0.05) (Table 5). The multivariate model additionally included age, sex, BMI, smoking status, hypertension, LDL‐C, HDL‐C, and eGFR to control for potential confounding. Inclusion of these covariates did not obviously affect the direction or statistical significance of the associations above (all VIF < 2.5).

**Table 5:** Multivariate logistic regression analysis results of NAFLD subjects for factors contributing to CHD.

**Indicator**	**B**	**Standard error**	**Wald**	**P**	**Odds ratio**	**95% confidence interval**
CK18‐M65 (U/mL)	0.001	0.001	4.918	0.027	1.001	1.000–1.002
CK18‐M30 (U/mL)	1.029	0.327	9.881	0.002	2.798	1.473–5.314
TC (mmol/L)	1.454	0.586	6.159	0.013	4.282	1.358–13.505
ALT (U/L)	0.206	0.090	5.224	0.022	1.229	1.031–1.469
AST (U/L)	0.587	0.274	4.575	0.032	1.798	1.053–2.079
HOMA‐IR	0.081	0.030	7.205	0.007	1.084	1.022–1.150

*Note:* Odds ratios (ORs) for CK18‐M30 and CK18‐M65 are expressed per 10 U/mL increase. Other continuous variables are expressed per unit increase in their respective scales.

### 3.9. ROC Curve Analysis Results

**Figure 4:**
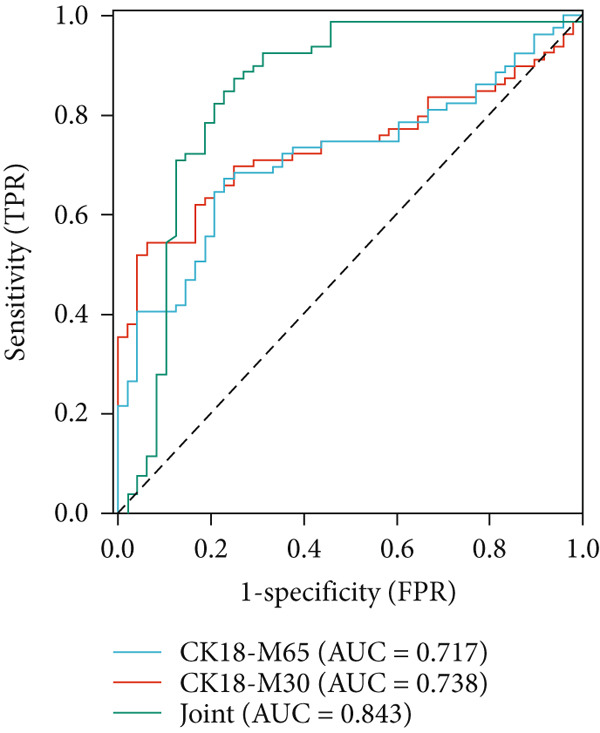
Analysis of ROC curves on value of CK18 for NAFLD subjects in predicting CHD.

ROC curve analysis was performed to evaluate the discriminatory ability of serum CK18‐M30 and CK18‐M65 for identifying NAFLD patients with concurrent CHD. Individually, CK18‐M65 and CK18‐M30 yielded AUCs of 0.717 (95% CI: 0.628–0.805) and 0.738 (95% CI: 0.652–0.823), respectively. The combined index, derived from a logistic regression model integrating CK18‐M65 and CK18‐M30, achieved an AUC of 0.843 (95% CI: 0.760–0.927), with sensitivity of 87.34% and specificity of 75.00%, at the optimal probability threshold determined by the maximum Youden index. DeLong′s test indicated that the combined model performed significantly better than CK18‐M65 and CK18‐M30 alone. Bootstrap internal validation (1,000 repetitions) yielded an optimism‐corrected AUC of 0.831, suggesting good internal stability. These results indicate that the combined CK18‐M65/M30 model provides strong discriminatory power for distinguishing NAFLD patients with prevalent CHD (Table 6 and Figure 4).

**Table 6:** Value of serum CK18 for NAFLD subjects in predicting CHD.

**Indicator**	**Area under the curve**	**Sensitivity**	**Specificity**	**Cut-off value**	**Youden index**	**p**	**95% confidence interval**
CK18‐M65 (U/mL)	0.717	67.09	77.08	107.83 U/mL	0.442	< 0.05	0.628–0.805
CK18‐M30 (U/mL)	0.738	54.43	93.75	158.71 U/mL	0.482	< 0.05	0.652–0.823
Combination	0.843	87.34	75.00	—	0.623	< 0.05	0.760–0.927

### 3.10. Calibration and Decision Curve Analysis Results

The CK18‐M30/M65 logistic regression model demonstrated satisfactory calibration (Figure 5a), indicating good agreement between predicted and observed risks. Decision curve analysis further confirmed the model′s clinical value (Figure 5b), showing higher net benefit compared with “treat‐all” or “treat‐none” strategies across a wide range of threshold probabilities (0.2–0.8).

Figure 5(a) Calibration plot (b) and decision curve analysis of CK18‐M30/M65 model.

**Figure:**
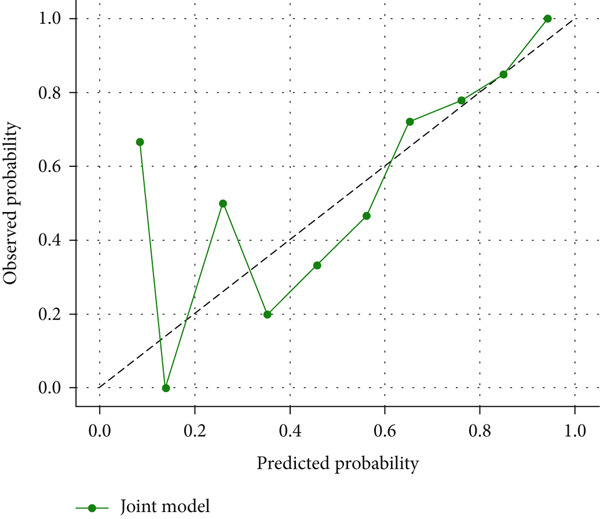
(a)

**Figure:**
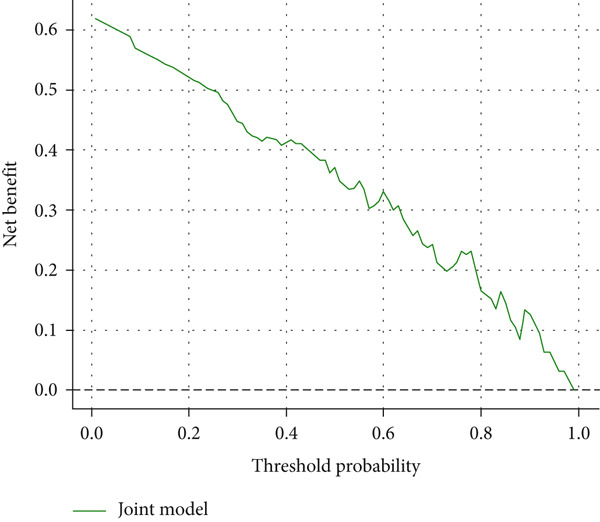
(b)

## 4. Discussion

NAFLD encompasses a spectrum of disorders characterized by excessive hepatocellular lipid accumulation. The disease is typically asymptomatic in its early stage, making early detection challenging without specific diagnostic testing. Consequently, NAFLD is often diagnosed only when it has progressed to advanced stages or when irreversible hepatic damage has occurred [17]. NAFLD has emerged as an important risk factor for atherosclerotic heart disease, which represents the leading cause of mortality among patients with NAFLD [18]. Individuals with NAFLD, particularly those with hepatic fibrosis or more advanced disease, exhibit a markedly increased prevalence of CVD. Moreover, NAFLD itself has been reported as an independent correlate of atherosclerotic CVD, and cardiovascular complications constitute a major contributor to the overall mortality of NAFLD patients [19]. The mechanistic link between NAFLD and CVD is multifactorial. First, patients with NAFLD frequently display insulin resistance, dyslipidemia, obesity, and other signs of metabolic syndrome, all of which are well‐established cardiovascular risk correlates. Second, NAFLD may contribute to cardiovascular dysfunction through systemic inflammatory responses, endothelial dysfunction, oxidative stress, and lipid metabolism variation [20]. Third, NAFLD is associated with a higher prevalence of atherosclerosis, cardiomyopathy, and arrhythmia. Specifically, hepatic insulin resistance, abnormal adipokine secretion, and chronic low‐grade inflammation in the liver may collectively promote atherosclerotic plaque formation and vascular injury. Thus, NAFLD not only predisposes individuals to CVD but may also actively participate in its pathogenesis. In this study, TC, ALT, AST, and HOMA‐IR levels were significantly increased in NAFLD patients with CHD, and these parameters were identified as independent factors associated with CHD. These findings suggest that abnormal glucose and lipid metabolism are closely linked to cardiovascular involvement in NAFLD. The detection of these biochemical indicators may therefore be useful for identifying NAFLD patients with coexisting cardiovascular conditions. Furthermore, when elevations in TC, ALT, AST, and HOMA‐IR are observed, combining biochemical markers with clinical and imaging assessments may help distinguish patients at higher likelihood of concurrent cardiovascular complications, thereby supporting timely clinical management to improve outcomes.

Similar to CVD, NAFLD represents a form of end‐organ damage caused by metabolic dysregulation, typically involving central obesity, hyperlipidemia, and hyperglycemia. NAFLD often precedes Type 2 diabetes and metabolic syndrome, and its bidirectional relationship with CVD demonstrates that NAFLD may be closely associated with cardiovascular involvement [21]. CK18, a Type I cellular keratin, is mainly expressed in epithelial monolayers and forms heteropolymers with Keratin 8, its filamentous partner and a member of the intermediate filament gene family. CK18 exists mainly in two measurable serum fragments: M30, a caspase‐cleaved fragment generated during apoptosis, and M65, the intact full‐length fragment released during both apoptosis and necrosis. Under physiological conditions, apoptosis contributes to hepatocyte turnover and maintenance of hepatic homeostasis. However, under pathological stress, such as infection, steatosis, or alcohol exposure, excessive hepatocyte apoptosis results in elevated release of CK18 fragments (M30 and M65) into the circulation [22]. In clinical practice, serum CK18‐M65 and CK18‐M30 level concentrations have been employed for diagnostic assessment and disease activity evaluation across a range of liver disorders, as well as in oncology research for tumor monitoring and therapeutic response evaluation [23]. CK18‐M65 demonstrates greater diagnostic relevance in advanced fibrosis compared with early‐stage disease (S0–S1). Persistent hepatocyte apoptosis has been reported to associate with fibrotic remodeling and worsening hepatic pathology [24]. In the present study, both CK18‐M65 and CK18‐M30 levels were significantly elevated in NAFLD patients, particularly among those with CHD, where they were identified as independent factors associated with CHD presence. Consistent with previous reports, NAFLD patients exhibited markedly higher CK18 levels than those with simple steatosis, and CK18 correlated positively with ALT, supporting its utility as a biochemical indicator of hepatocellular injury [25]. Mechanistically, CK18 release reflects both hepatocyte apoptosis and necrosis, with M30 representing caspase‐mediated apoptosis, and M65 reflecting total cell death (apoptotic + necrotic). This distinction is particularly valuable for differentiating NASH from simple steatosis and for assessing disease severity [26]. Collectively, these findings suggest that CK18 may serve as a promising biomarker reflecting disease activity and hepatic injury status in NAFLD and NASH, while also implicating a potential mechanistic link between hepatocyte apoptosis and cardiovascular involvement in this metabolic disorder.

Endothelial cell apoptosis contributes to endothelial dysfunction or hypertension, whereas cardiomyocyte apoptosis is closely associated with aging and chronic cardiac overload [27]. Serum CK18‐M30 levels have been reported to correlate strongly with left ventricular diastolic dysfunction in obese adolescents [28]. Moreover, CK18‐M30 expression peaks approximately 24 h after the onset of acute myocardial infarction, reflecting the extent of coronary artery injury and the severity of disease [29]. These findings are consistent with the current study, in which serum CK18‐M30 levels were significantly elevated in NAFLD patients with CHD. Elevated CK18‐M65 level has also been identified as an indicator associated with cardiac metabolic alterations. Similarly, continuous increases in both CK18‐M30 and CK18‐M65 levels have been observed in NAFLD patients with Type 2 diabetes mellitus, supporting their relevance to systemic metabolic disturbance and cardiovascular involvement [30]. In agreement with previous reports, our study demonstrated significant differences in CK18‐M30 and CK18‐M65 levels between NAFLD patients with and without CHD. These results suggest that elevated serum CK18 not only reflects hepatocyte injury and apoptosis but also may be linked to systemic inflammatory state and metabolic dysregulation, both of which are key drivers of the development and progression of CVD [31]. Taken together with the present findings, increased CK18 levels in NAFLD patients may indicate a stronger cardiometabolic burden mediated through inflammation and metabolic imbalance. Furthermore, the combined measurement of CK18‐M65 and CK18‐M30 demonstrated strong discriminatory ability for identifying NAFLD patients with concurrent CHD in this study, highlighting their potential diagnostic utility as complementary biomarkers. Given the close relationship between NAFLD and CVD, and the sensitivity of CK18 to hepatocellular injury and inflammatory signaling, serum CK18 may provide a novel and practical approach for identifying NAFLD patients with coexisting cardiovascular complications, thereby supporting improved clinical evaluation and management.

Notably, pathway analysis provided mechanistic insights into the role of CK18 in linking hepatic injury to cardiovascular dysfunction. Insulin resistance in NAFLD initiates lipid accumulation and oxidative stress, leading to hepatocyte damage and CK18 release. This is supported by the KEGG insulin signaling and PPAR pathways, which indicate that disruptions in lipid metabolism and energy homeostasis promote hepatic damage [32]. Additionally, the TNF and NF‐*κ*B signaling pathways highlight the contribution of systemic inflammation originating from the liver (“hepatic‐inflammatory axis”) to endothelial dysfunction, a hallmark of atherosclerosis and CHD [33]. Therefore, CK18 works as a marker of this inflammation‐apoptosis cascade associated with NAFLD progression. Moreover, activation of caspase‐dependent apoptosis via endoplasmic reticulum stress and mitochondrial dysfunction further underscores the importance of CK18 as a biomarker linking hepatocyte injury, systemic metabolic dysfunction, and CVD risk [34]. These findings collectively suggest that CK18 may represent a biologically plausible indicator of hepatic–cardiac interaction, where NAFLD‐associated hepatocyte injury is associated with systemic inflammation and increased cardiovascular susceptibility. Clinically, serum CK18 shows promise for diagnostic discrimination and early risk stratification in NAFLD patients with potential cardiovascular complications.

Several limitations should be acknowledged. First, the relatively small sample size needs validation in larger cohorts. Second, the cross‐sectional design precludes causal inference, and longer follow‐up is needed to determine the prognostic value of CK18. Third, exclusion of patients with diabetes and hypertension reduced confounding but limits generalizability; future studies should include these populations. Additionally, partial correlation and sensitivity analyses excluding transaminases were not performed; larger studies are warranted to clarify these interrelationships and confirm the independence of CK18 as a diagnostic marker. Moreover, CHD was assessed as a binary outcome rather than by angiographic severity or lesion burden, which may underestimate the association between CK18 and cardiovascular pathology. Future studies incorporating quantitative measures of CHD burden are needed to validate the relationship between CK18 levels and disease severity. Finally, the incremental value of CK18 beyond conventional cardiovascular risk factors (age, sex, lipids, blood pressure, and smoking) was not assessed and should be explored in future prospective models.

## 5. Conclusion

In conclusion, serum levels of CK18‐M30 and CK18‐M65 are significantly elevated in patients with NAFLD and show strong associations with the presence of CVD. Serum CK18 holds potential as a diagnostic and risk‐stratification biomarker for cardiovascular involvement in NAFLD. Future research should focus on interventions targeting metabolic and inflammatory pathways to mitigate hepatocyte apoptosis and thereby CVD risk in NAFLD patients. In addition, longitudinal studies are warranted to further validate CK18 as a prognostic biomarker for long‐term cardiovascular outcomes in NAFLD patients.

## Ethics Statement

This study was reviewed and approved by the Ethics Committee of Wuxi Xishan People′s Hospital (Approval No. ZW2022.105). All participants were informed about the purpose and procedures of the study and provided written informed consent prior to enrollment. The study was conducted in accordance with the Declaration of Helsinki and relevant local ethical guidelines.

## Disclosure

All authors gave final approval of the version to be published and agreed to be accountable for all aspects of the work.

## Conflicts of Interest

The authors declare no conflicts of interest.

## Author Contributions

All authors contributed to the data analysis and the writing of this paper.

## Funding

No funding was received for this manuscript.

## Supporting information


**Supporting information** Additional supporting information can be found online in the Supporting Information section. Table S1 lists the expression summary for ligand–receptor pairs. Figure S1 shows KEGG pathway analysis revealing that CK18 elevation is linked to multiple pathological processes in NAFLD.

## Data Availability

The datasets generated and/or analyzed during the current study are not publicly available due to institutional privacy restrictions but are available from the corresponding author on reasonable request.
