# Supplementary material for: From the Liver to Heart in Nonalcoholic Fatty Liver Disease: Single‐Cell and Serum Evidence for Cytokeratin 18 in Predicting Cardiovascular Risk
Source: Cardiovasc Ther. 2026 Jan 15;2026:3055531. doi: 10.1155/cdr/3055531 (PMC12808931; doi:10.1155/cdr/3055531)
Supplement: Supplementary file 1 — Supporting information Additional supporting information can be found online in the Supporting Information section. Table S1 lists the expression summary for ligand–receptor pairs. Figure S1 shows KEGG pathway analysis revealing that CK18 elevation is linked to multiple pathological processes in NAFLD. [file CDR-2026-3055531-s001.docx]

Table S1. Expression summary for ligand-receptor pairs

| Gene | Cell group | Mean expression (log-normalized) |
| --- | --- | --- |
| TGFB1 | Hep_KRT18_high | 0.0015 |
| TGFB2 | Hep_KRT18_high | 0.0000 |
| TGFB3 | Hep_KRT18_high | 0.0015 |
| IL6 | Hep_KRT18_high | 0.0015 |
| CXCL10 | Hep_KRT18_high | 0.034 |
| TGFBR2 | Cardiac | 0.081 |
| IL6R | Cardiac | 0.010 |
| CXCR3 | Cardiac | 0.0000 |


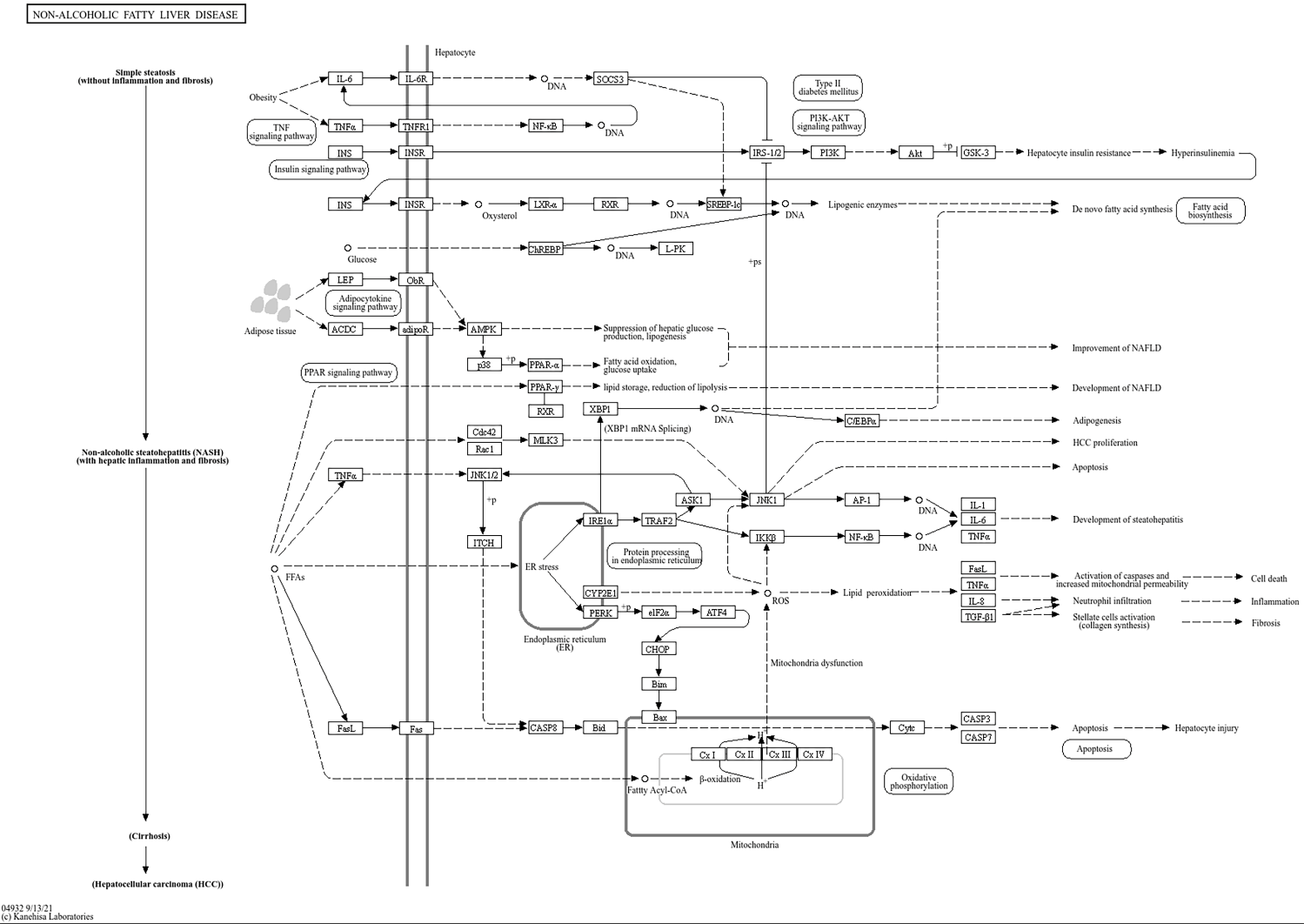


Figure S1. KEGG pathway analysis revealed that CK18 elevation is linked to multiple pathological processes in NAFLD.
